# Investigation of the Electrical Properties of Microtubule Ensembles under Cell-Like Conditions

**DOI:** 10.3390/nano10020265

**Published:** 2020-02-05

**Authors:** Aarat P. Kalra, Sahil D. Patel, Asadullah F. Bhuiyan, Jordane Preto, Kyle G. Scheuer, Usman Mohammed, John D. Lewis, Vahid Rezania, Karthik Shankar, Jack A. Tuszynski

**Affiliations:** 1 Department of Physics, University of Alberta, 11335 Saskatchewan Dr NW, Edmonton, AB T6G 2M9, Canada; aarat@ualberta.ca (A.P.K.); preto@ualberta.ca (J.P.); jackt@ualberta.ca (J.A.T.); 2 Department of Electrical and Computer Engineering, University of Alberta, 9107–116 St, Edmonton, AB T6G 2V4, Canada; sdpatel@ualberta.ca (S.D.P.); abhuiyan@ualberta.ca (A.F.B.); scheuer@ualberta.ca (K.G.S.); 3 Department of Physical Sciences, MacEwan University, Edmonton, AB T5J 4S2, Canada; mohammedu@mymacewan.ca (U.M.); rezaniav@macewan.ca (V.R.); 4 Department of Oncology, University of Alberta, Edmonton, AB T6G 1Z2, Canada; jdlewis@ualberta.ca

**Keywords:** microtubules, bioelectricity, bionanowires, neuronal charge storage, impedance spectroscopy, cytoskeleton

## Abstract

Microtubules are hollow cylindrical polymers composed of the highly negatively-charged (~23e), high dipole moment (1750 D) protein α, β- tubulin. While the roles of microtubules in chromosomal segregation, macromolecular transport, and cell migration are relatively well-understood, studies on the electrical properties of microtubules have only recently gained strong interest. Here, we show that while microtubules at physiological concentrations increase solution capacitance, free tubulin has no appreciable effect. Further, we observed a decrease in electrical resistance of solution, with charge transport peaking between 20–60 Hz in the presence of microtubules, consistent with recent findings that microtubules exhibit electric oscillations at such low frequencies. We were able to quantify the capacitance and resistance of the microtubules (MT) network at physiological tubulin concentrations to be 1.27 × 10^−5^ F and 9.74 × 10^4^ Ω. Our results show that in addition to macromolecular transport, microtubules also act as charge storage devices through counterionic condensation across a broad frequency spectrum. We conclude with a hypothesis of an electrically tunable cytoskeleton where the dielectric properties of tubulin are polymerisation-state dependent.

## 1. Introduction

Microtubules (MTs) are cylindrical polymers composed of the heterodimers of protein α, β- tubulin that play a variety of well-recognised intracellular roles, such as maintaining the shape and rigidity of the cell, aiding in positioning and stabilisation of the mitotic spindle for allowing chromosomal segregation, acting as ‘rails’ for macromolecular transport and forming cilia and flagella for cell movement. Since the tubulin dimer possesses a high negative electric charge of ~23e and a large intrinsic high dipole moment of approximately 1750 D [1,2], MTs have been implicated in electrically-mediated biological roles [3,4,5,6]. They have been modelled as nanowires capable of enhancing ionic transport [7,8], and simulated to receive and attenuate electrical oscillations [4,9,10,11]. In solution, MTs have been shown to align with applied electric fields [2,12,13,14,15,16]. Recently, MTs have also been modelled as the primary cellular targets for low-intensity (1–2 V), intermediate-frequency (100–300 kHz) electric fields termed TTFields (tumour-treating electric fields) that inhibit cancer cell proliferation, in particular glioma [17,18,19]. Indeed, MTs have been reported to decrease buffer solution resistance [12,13], leading to a conductance peak at frequencies close to the TTField regime [20]. While these studies show that MTs are highly sensitive to external electric fields, answers to the questions ‘How do MTs effect a solution’s capacitance?’ and ‘What is the capacitance of a single MT?’ are still elusive and crucial to the determination of the dielectric properties of living cells. The tubulin concentration in mammalian cells varies in the micromolar range (~10–25 μM) [21,22]. In vitro, polymerizing tubulin at such high concentrations can lead to the formation of entangled networks, confounding quantification of the individual MT response to electric fields. Electro-rotation, di-electrophoresis and impedance spectroscopy are thus performed using low concentrations of tubulin, in the nanomolar regime, to enable robust observation of individual MTs.

MT formation and stability are known to be optimal in buffers with ionic strength between 80 and 100 mM [23,24]. A background of BRB80 (which consists of 80 mM PIPES, 2 mM MgCl_2_ and 0.5 mM EGTA, containing ~46 mM PIPES ^2–^, ~36 mM PIPES^–^, ~68 mM Cl^–^, ~160 mM K^+^, and ~2 mM Mg^2+^ [2]), is thus used to study the dynamics and mechanical properties of MTs. To study their electrical properties however, the usage of such high ionic-strength solutions has historically been problematic because any dielectric attenuation caused by MTs is overwhelmed by the noise and high conductivity from the background. In the low-frequency regime (1–100 kHz), two approaches have thus far been used to estimate the dielectric properties of MTs and tubulin. One is to electrically observe low concentrations of MTs (tubulin concentration in the nanomolar regime) in the presence of low ionic strengths [12,13,20,25,26]. Such studies overlook the intrinsic ionic concentration of mammalian cytosol, which varies between 200 to 500 mM depending on the cell type [27,28]. Another approach to electrically interrogate MTs is to dry them: the conductivity of the buffer is nullified by evaporation, leaving polymeric tubulin behind [29,30]. In a physiological situation however, MTs are solvated by the highly conductive and noisy cytosol. 

Here, we report on our efforts overcome the barrier posed by a high ionic strength by performing electrochemical impedance spectroscopy (EIS) on cellular concentrations of tubulin. We have been able to successfully observe differences in impedance using a background of BRB80 itself. Surprisingly, we find that MTs increase the solution capacitance of BRB80 whereas free tubulin does not, implicating a difference in electrical properties based only on the morphology of this protein solute. We also report a ‘reversal’ in the resistive behaviour of MTs compared to BRB80, with a reduction in solution resistance peaking in the 20–60 Hz region, a finding consistent with recent reports showing that polymerised tubulin quasi-resonantly responds to electric oscillations at ~39 Hz [9,10]. Using an equivalent circuit model for MTs, we experimentally determine the capacitance and resistance of the MT network to be 1.27 × 10^−5^ F and 9.74 × 10^4^ Ω respectively, at physiological concentrations of tubulin. Our values indicate that the polymerisation of tubulin into MTs alters the spatial and temporal charge distribution, altering the electrical properties through charge storage in the cell. 

## 2. Materials and Methods 

### 2.1. Tubulin Reconstitution

Fluorescently labelled tubulin solution was prepared using previously published protocols [20]. Notably, tubulin stock powders that were devoid of MAPs were purchased. Lyophilised unlabelled tubulin powder (Cytoskeleton Inc, Denver, CO, USA; T240) was reconstituted in BRB80 supplemented with 1 mM GTP (guanosine triphosphate; Cytoskeleton Inc, Denver, CO, USA; BST06) and mixed with tubulin labelled with a rhodamine-based ester (Cytoskeleton Inc, Denver, CO, USA; TL590m) in a final labelling ratio of 1:15. Aliquots were snap-frozen and stored at −80 °C. 

### 2.2. MT Polymerisation and Stabilisation

MT polymerisation was performed by incubating 45.45 μM tubulin aliquots in a 37 °C water bath for 30 minutes. BRB80 solution was heated alongside tubulin during the first 15 minutes of polymerisation. Subsequently, BRB80 was incubated at room temperature, and paclitaxel solution (Cytoskeleton Inc, Denver, CO, USA; TXD01; 2 mM stock) was thawed at room temperature alongside it. After 30 minutes of tubulin polymerisation brought to completion, 100 μL of BRB80 was added to 5 μL of 2 mM paclitaxel (BRB80T). For preparing 0.222, 2.225 and 22.225 μM MTs the above solution was added in different volumes to polymerised tubulin, as shown in Table 1. For preparing the BRB80T background for impedance measurements, 45 μL of this solution was added to 45 μL of BRB80. 

For tubulin stabilisation, 2 μL of 5 mM colchicine stock solution (Sigma-Aldrich, St. Louis, MO, USA; C9754; 5 mM in DMSO) was added to 100 μL BRB80 (BRB80C). Subsequently, a similar solution to BRB80T was prepared using colchicine. For preparing 0.222, 2.225 and 22.225 μM free tubulin solutions, the above solution was added in different volumes to free tubulin solutions, as shown in Table 1. For preparing BRB80C, 45 μL of this solution was added to 45 μL of BRB80. 

### 2.3. Fluorescence Imaging of MTs 

Imaging was performed on a Zeiss Examiner.Z1 microscope using a Hamamatsu (Hamamatsu City, Japan) EMCCD C9100 camera, a Zeiss (Oberkochen, Germany) plan-Apochromat 1.4 NA 63x lens. After pipetting MT solution (2–5 μL) onto a glass slide (VWR 48382-173) a coverslip (VWR 48393-070) was placed on the solution, allowing it to spread. The microscope used an EXFO X-Cite 120 fluorescence source and excitation and emission filters of 535 nm and 610 nm, respectively. Exposure times between 50 ms and 300 ms were used for imaging to validate the presence of MTs.

### 2.4. Electrode Design and Device Construction 

Each ‘plate’ in the parallel-plate contact device was formed by FTO (fluorine-doped tin oxide)-coated glass slides (Sigma Aldrich, St. Louis, MO, USA; 735140). The slides were cleaved to dimensions of 1.5 mm × 10 mm × 50 mm for the upper contact and 1.5 mm × 27 mm × 50 mm for the lower contact. The cleaving dimensions were set using 3D printed devices that were placed as holders (The Shack, University of Alberta; Figure S1 in Supplementary Materials). The slides were ultrasonicated and subjected to reactive ion etching (RIE) using a 5-minute exposure to oxygen plasma (Oxford Instruments, Abingdon, UK; NGP80) to remove surface particulate matter. A 70-μm thick double-sided tape was used as a spacer, which formed a chamber of dimensions 3 mm × 1.25 cm × 70 μm. The top electrode was placed using a separate 3D-printed holder device (Figure S1). Once the device was constructed using the above protocol, solution was perfused into the chamber using a pipette and a filter paper for suction. We used flat copper electrode clips in a three-electrode configuration to connect to our capacitor device. The counter electrode was connected to the lower electrode, and the working and reference electrodes were connected to the top electrode of our device. 

### 2.5. Impedance Measurements

Experiments were conducted using Electrochemical Impedance Spectroscopy (EIS) on a Zahner Zennium impedance analyser. The parallel-plate contact device was placed into the 3D-printed holder for stabilisation (Figure S1). The contacts from the machine were connected to the parallel-plate device using flat-faced copper alligator clips. A three-electrode configuration was used: The counter electrode was attached to the lower contact of the parallel-plate device, whereas the working electrode was attached to the upper contact with the reference electrode orthogonally clipped onto the clip of the working electrode. Within the Thales Z3.04 environment, the potentiostat mode was ON; the stabilisation delay was set to 1 s, the rest potential drift tolerance was set to 250 μV, V_rms_ was set to 5 mV. Solutions were perfused into the experimental chamber using a micropipette tip at one opening, and a filter paper at the other opening for suction, similar to protocols used for Total Internal Reflection Fluorescence (TIRF) microscopy [31]. The frequency range of the EIS measurement was set from 4 MHz to 1 Hz and data were subsequently collected. 

### 2.6. Data Analysis 

MT and tubulin samples were analysed using data from five to seven days of experiments. Each day consisted of three to seven solutions for each concentration being tested, with one frequency sweep per solution. Readings of each sweep were saved as a csv file, and next sample was loaded by solution exchange method. Water was run as the first solution for each day of experiments. BRB80T was run prior to MT solutions, and BRB80C were run prior to the free tubulin containing solutions. MT- and free tubulin-containing solutions were run on separate days, in increasing order of concentration. MATLAB (The Mathworks; Natick, MA, USA) scripts were used for data analysis. Fitting to the real and imaginary components of impedance was performed using the function lsqnonlin. Initial guess values for the MT network resistance and capacitance were 10^5^ F and 10^−5^ Ω, respectively, based on visual inspection of raw data. The initial guess values for the nominal series resistor, R_H_, were set at 1.78, 0.6 and 0.4 Ω with tubulin concentrations of 0.222, 2.222 and 22.222 μM, respectively. The 95% confidence intervals were determined using the function nlparci. Error propagation was performed assuming no relationship between various days of data collection.

## 3. Results

### 3.1. Validation of Parallel-Plate Contact Device to Measure Dielectric Properties of Physiologically Relevant Ionic Solutions 

**Figure 1:**
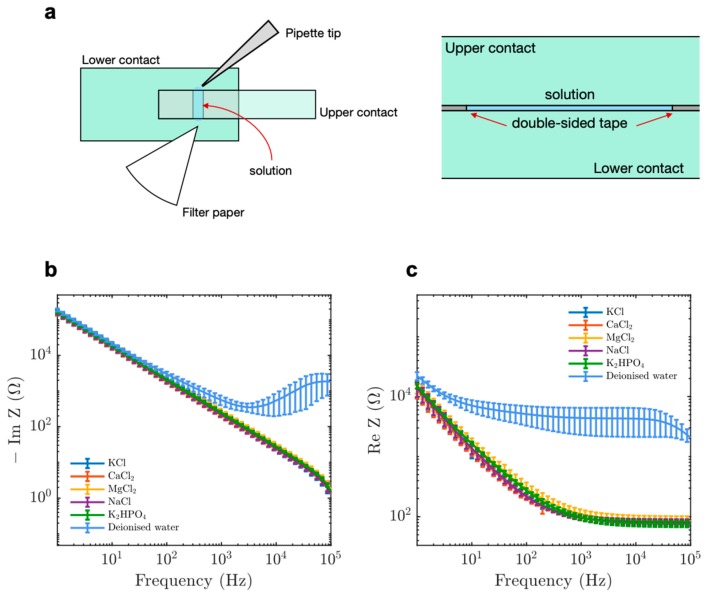
A parallel-plate contact device to measure the impedance properties of microtubules (MTs) compared to tubulin. The operation of the parallel plate device showing (**a**) top view (left) and side view (right). The upper and lower contacts, double-sided tape and solution are labelled in green, grey and blue, respectively. (**b**) Imaginary component of impedance for electrolytic solutions at 100 mM and de-ionised water. (**c**) Real component of impedance for electrolytic solutions at 100 mM and de-ionised water. Data display average values collected between 15 and 21 times. Error bars represent standard deviation.

To determine the differences in the dielectric properties of solution caused by the presence of MTs, we first aimed to create an electrode geometry that would be experimentally robust and easily modelled. We fabricated an FTO-coated parallel-plate contact device (Figure 1a), which allowed EIS using a solution-exchange method.

We started by performing EIS on electrolytes found in the cytosol and observed that the imaginary component of impedance became less negative as a function of applied input frequency (Figure 1b). The total impedance of our system was given by:

*Z* = *r_c_* + *r_s_*/(1 + (*r_s_ωC*)^2^) + *j*(*ωL_c_* – (*r_s_*^2^*ωC*)/(1 + (*r_s_ωC*)^2^)), (1)

Here, *Z* is the impedance, *ω* is the angular frequency (given by *2πf* where *f* is the input voltage frequency), *C* is the system capacitance, *L_c_* is the cable inductance, *r_s_* and *r_c_* are the solution and cable resistances respectively. We also observed a decrease in the real component of impedance as a function of input frequency (Figure 1c). Such a trend is expected from Warburg impedance [32,33] and is in accordance with the equation:

*Z_complex_* = (A*_ω_*)/√*ω* + (A*_ω_*)/(*j*√*ω*), (2)

Here, *Z_complex_* is the complex impedance and A*_ω_* is the Warburg coefficient. Our circuit simplifies to the equation below if we ignore the effect of cable inductance *ωL_c_*, at frequencies below 10^5^ Hz:

*Z* = *r_c_* – *j*/*ωC*, (3)

Our results using various electrolytes emulated previous data [34,35,36] and validated the experimental setup for further analysis. 

### 3.2. The Effect of Microtubule Networks on Solution Capacitance at Physiologically Relevant Conditions

**Figure 2:**
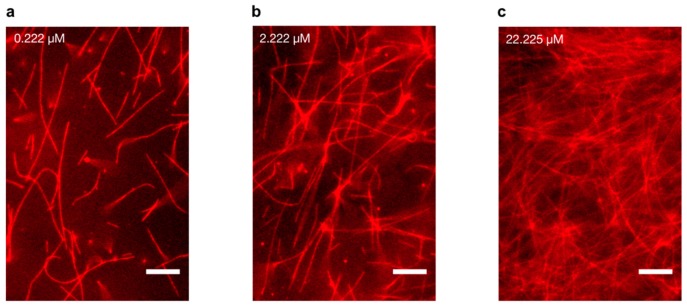
Microtubule imaging at different tubulin concentrations. Polymerisation was performed using 45 µM tubulin, and MTs were stabilised with 50 µM paclitaxel, and subsequently diluted to a final concentration of (**a**) 0.222 µM tubulin (**b**) 2.222 µM tubulin (**c**) 22.225 µM tubulin, respectively. Scale bars represent 10 μm.

We reconstituted and polymerised fluorescent tubulin from a stock of 45.45 μM tubulin solution (Materials and Methods). MTs were stabilised using 50 μM paclitaxel [37,38] and imaged using an epi-fluorescence microscope. On diluting MT concentration across three orders of magnitude (0.222, 2.222 and 22.225 μM tubulin), we observed that while individual MTs at low concentrations were separated by large distances, those at cellular concentrations formed enmeshed networks reported previously (Figure 2a–c) [39]. Such interconnected MT networks are utilised by molecular motors for long-range macromolecular transport [40,41]. Here, their presence demonstrated successful MT polymerisation for electrical characterisation. 

**Figure 3:**
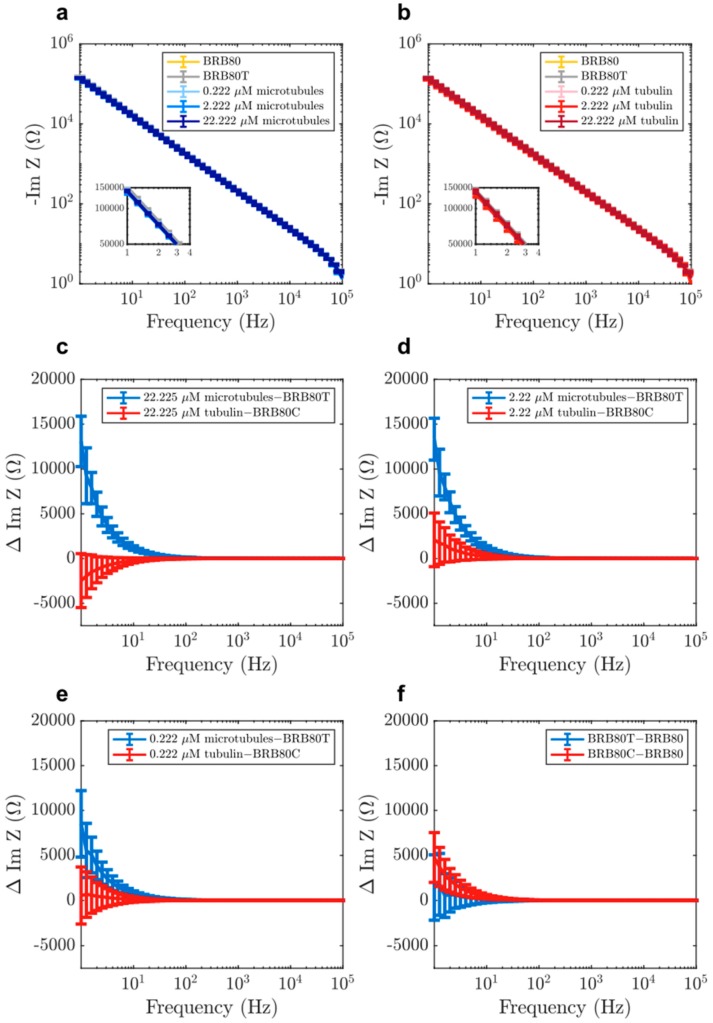
Examples of raw values of imaginary component of impedance in solutions containing (**a**) MTs and (**b**) tubulin, for the purpose of displaying typical impedance values. Mean differences in the imaginary component of impedance as a function of input AC (alternating current) frequency at total tubulin concentrations of (**c**) 22.225 µM (n = 22 experiments for tubulin, n = 21 for MTs), (**d**) 2.222 µM (n = 35 experiments for tubulin, n = 49 for MTs) (**e**) 0.222 µM (n = 35 experiments for tubulin, n = 49 for MTs), (**f**) comparison of the effects of paclitaxel (BRB80T) and colchicine (BRB80C, n = 49 experiments for BRB80T, n = 35 for BRB80C, n = 84 experiments for BRB80). Error-bars represent standard deviation.

We performed EIS on BRB80, BRB80T (BRB80 supplemented with 50 μM paclitaxel; background for all MT-containing solutions), and MT-containing solutions in increasing order of concentration (Figure 3a,b). We subtracted impedance values obtained for BRB80T alone from those in MT-containing solutions to determine the MT contribution to impedance. Our results showed that with an increasing MT concentration, the value of imaginary impedance became more negative, resulting in positive impedance differences (Figure 3c–f). This effect was greatest at the cell-like 22.225 µM tubulin concentration, with impedance differences lowering in magnitude with increasing input frequency (Figure 5a). Experiments with unpolymerised tubulin at the same concentrations were performed using the identical procedure, but using BRB80C (BRB80 was supplemented with 50 μM colchicine) as a background, to prevent MT nucleation [42,43]. Results with unpolymerised tubulin did not show an appreciable deviation from zero at any concentration (Figure S5a). The above results suggest that polymerisation of tubulin into MTs alters their ensemble electrical properties, increasing the solution’s capacitance on forming MTs and their networks. An increase in the solution’s capacitance because of MTs has previously been modelled, [7,44,45] indicating an increase in charge storage as free tubulin polymerises. 

### 3.3. The Effect of Microtubule Networks on Solution Resistance at Physiologically Relevant Conditions 

**Figure 4:**
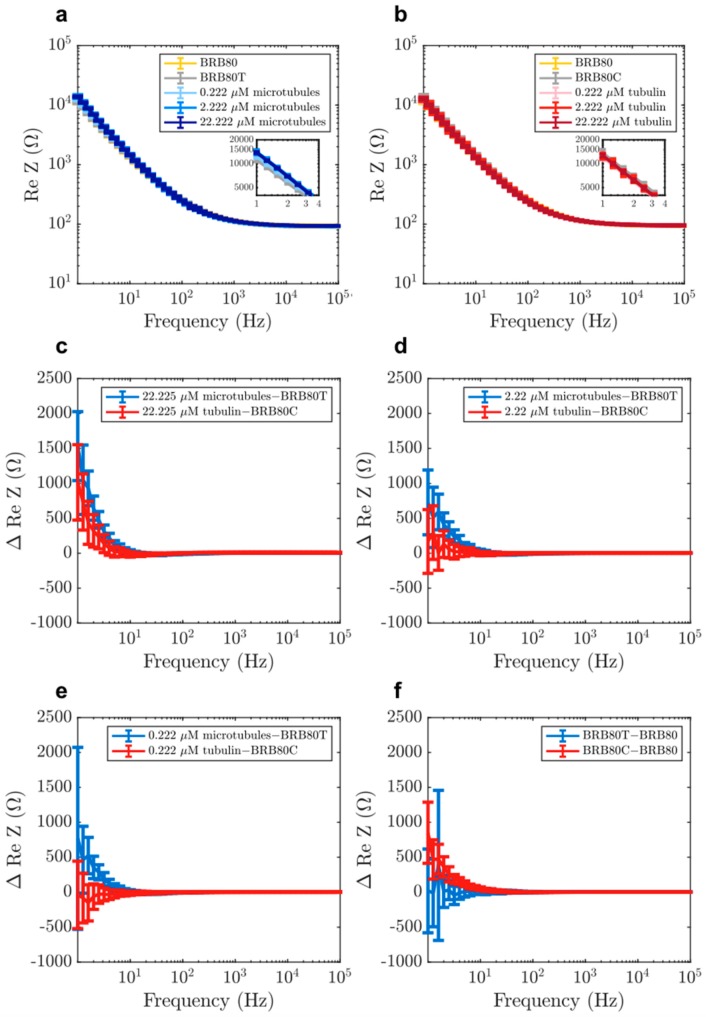
Examples of raw values of real component of impedance in solutions containing (**a**) MTs and (**b**) tubulin, for the purpose of displaying typical impedance values. Mean differences in the real component of impedance as a function of input AC frequency at total tubulin concentrations of (**c**) 22.225 µM (n = 22 experiments for tubulin, n = 21 for MTs), (**d**) 2.222 µM (n = 35 experiments for tubulin, n = 49 for MTs) (**e**) 0.222 µM (n = 35 experiments for tubulin, n = 49 for MTs), (**f**) comparison of the effects of paclitaxel (BRB80T) and colchicine (BRB80C, n = 49 experiments for BRB80T, n = 35 for BRB80C, n = 84 experiments for BRB80). Error-bars represent standard deviation.

**Figure 5:**
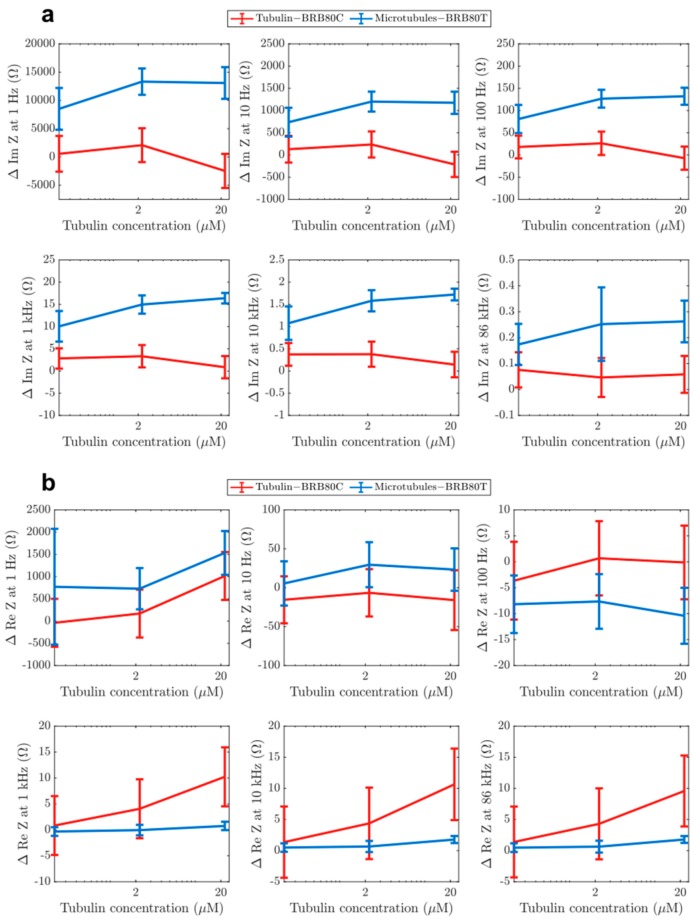
Graphs showing differences in the (**a**) imaginary from Figure 3 and (**b**) real component of impedance from Figure 4 as a function of tubulin concentration at input AC frequencies of 1 Hz, 10 Hz, 100 Hz, 1 kHz, 10 kHz and 86 kHz. Graphs display average values. Error-bars represent standard deviation.

**Figure 6:**
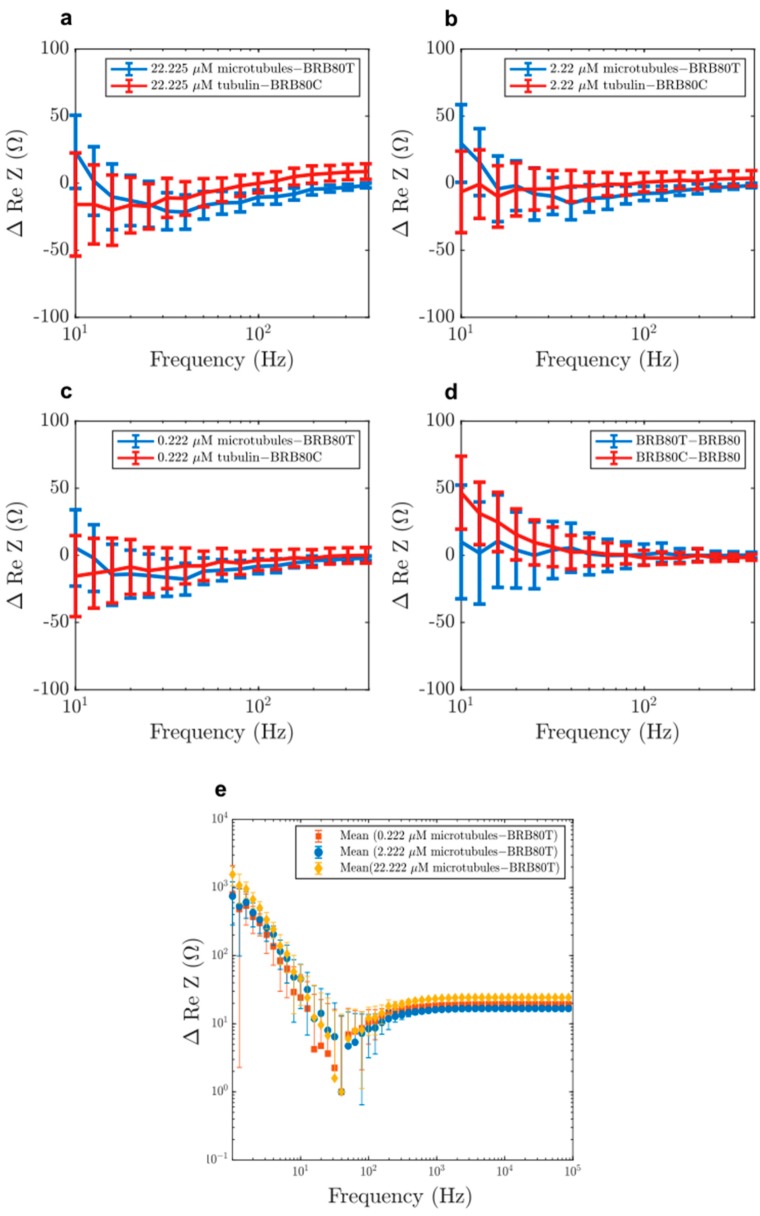
Zoomed in view of the mean differences in the real component of impedance as a function of decreasing input AC frequency at total tubulin concentrations of (**a**) 22.225 µM, (**b**) 2.222 µM, (**c**) 0.222 µM, (**d**) comparison of the effect of paclitaxel and colchicine on impedance. (**e**) A logarithmic plot obtained by translating the graphs (a), (b) and (c) upwards. The translation is performed by adding (1+minimum MT solution resistance) to the resistance of each MT concentration. A resistance reversal between 20–60 Hz is observed, with a peak at 39 Hz for the 22.225 µM concentration. Error-bars represent standard deviation.

Next, we investigated the differences between MTs and tubulin in the real component of impedance (solution resistance). Previous studies using nanomolar tubulin concentrations and low ionic strengths (1–12 mM) have indicated that MTs enhance charge-transport in solutions [13,20,46]. To evaluate if this observation held true at physiologically relevant tubulin concentrations and at higher ionic strengths, we also analysed the real component of impedance. Addition of both MTs and tubulin generally led to an increase in solution resistance (Figure 4a–f), with MTs having a higher resistance at low frequencies (1–20 Hz) compared to unpolymerised tubulin. Unexpectedly, a ‘reversal’ of this behaviour was observed at higher frequencies as MTs began to lower the solution resistance compared to tubulin (Figure 5b). The reversal took place gradually between 10 and 300 Hz (Figure 6a–d), with a peak between 20 and 60 Hz (Figure 6e). Interestingly, within this range, we also found that the addition of MTs lowered solution resistance compared to background buffer BRB80T. 

Such a reversal in resistance between microtubules and tubulin has not been reported before. Because the extent of this reversal decreased with decreasing concentration, this result also displays the utility of our ‘cell-like’ approach. Our results are consistent with predictions of an increase in solution conductance at ~39 Hz [9,10], which have been hypothesised to arise from oscillatory ionic movement across the MT lattice through nanopores formed between adjacent tubulin dimers (Figure 9a). 

It is worth noting that this region falls within the gamma frequency regime (20–60 Hz), implicating such quasi-resonant phenomena as a possible explanation for the source of low frequency intraneuronal electrical oscillations. No such reversal was observed for the corresponding frequency range in the imaginary impedance values (Figure S4).

### 3.4. The Microtubule Network as an RC Circuit in Parallel 

Our next aim is to quantify the resistance and capacitance of the microtubule network. The slope of approximately negative unity on the impedance difference curve suggested that the microtubule network resulted in the addition of a capacitive element to the solution. We examined several combinations but a parallel RC (resistor-capacitor) circuit to represent the entire MT network provided the best fit to observed curves.

**Figure 7:**
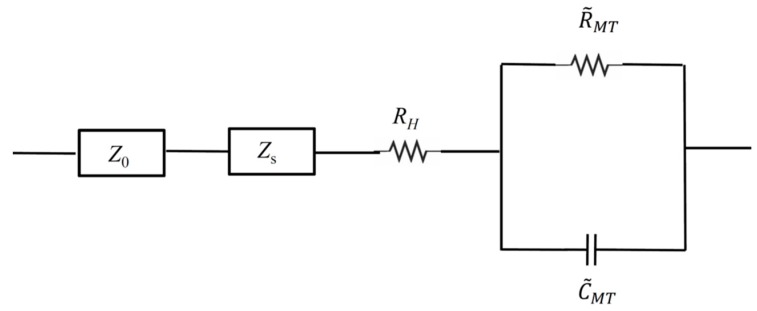
The equivalent electrical circuit model representing the microtubule network as a parallel RC circuit, with network resistance *R_MT_* and capacitance *C_MT_*. The external element has impedance *Z_0_*, while solution has impedance *Z_s_*. *R_H_* is the small constant resistance that is ascribed to small fraction of unpolymerised tubulin that is present in MT containing solutions.

We modelled the impedance caused by external circuit elements and BRB80T as *Z_o_* and *Z_s_* respectively, as shown in Figure 7. The net impedance of the background BRB80T was thus given by:

Z_buffer_ = Z_0_ + Z_s_, (4a)

Denoting the impedance, resistance and capacitance of the entire MT network by *Z_MT_*, *R_MT_* and *C_MT_* respectively, the impedance for the circuit with MTs is given by:

*Z_MT+buffer_* = *Z*_0_ + *Z_s_* + *R_H_* + *Z_MT_* (4b)

where,

1/*Z_MT_* = 1/*R_MT_* + *jωC_MT_* (5)

Additionally, the impedance differences between solutions with and without MTs are given by:

*ΔZ* = *Z_MT+buffer_* – *Z_buffer_* = *R_H_* + *Z_MT_*, (6)

where

*Z_MT_*=*R_MT_*/(1 + (*ωC_MT_R_MT_*)^2^) – *j*(*ωC_MT_R_MT_*^2^)/(1 + (*ωC_MT_R_MT_*)^2^), (7)

**Figure 8:**
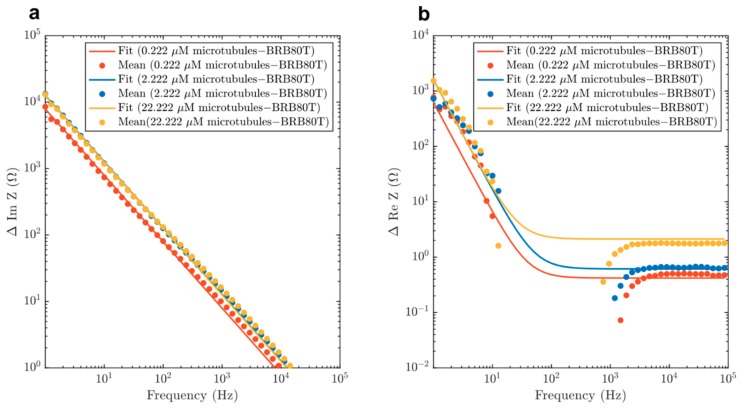
Mean differences of (**a**) imaginary and (**b**) real impedance curves for 0.222 µM, 2.222 µM and 22.222 µM, are fitted with the model described in Equation (6) and Figure 7. Fit parameters and confidence intervals are displayed in Table 2. The region between 1–100 Hz was not fit because of the negative differences in resistance from background BRB80T solutions.

We subsequently fit experimental impedance difference curves shown in Figure 3 and Figure 4 to real and absolute value of imaginary parts of *ΔZ* using *R_H_*, *R_MT_* and *C_MT_* as our fit parameters. Here, *R_H_* is a resistance ascribed to the nominal fraction of unpolymerised tubulin present in MT containing solutions. The fitted curves are displayed in Figure 8 and the optimal fit parameters are listed in Table 2 (see Materials and Methods for details). 

## 4. Discussion

Our measurements using a parallel plate contact device reveal interesting electrical properties of MTs at physiological concentrations. Unlike studies exposing MT-containing solutions to non-uniform electric fields [12,13,14,20], our device allowed robust quantification of electrical impedance in the presence of spatially uniform electric fields. Our results show that the addition of the MT network mimics a parallel RC element placed in series with the high-ionic strength solution, with a nonlinear dependence on MT number. Unpolymerized tubulin did not alter the capacitance significantly, indicating changes in electrical properties of tubulin as it polymerizes. 

### 4.1. The Physical Underpinnings of An Increased Capacitance 

**Figure 9:**
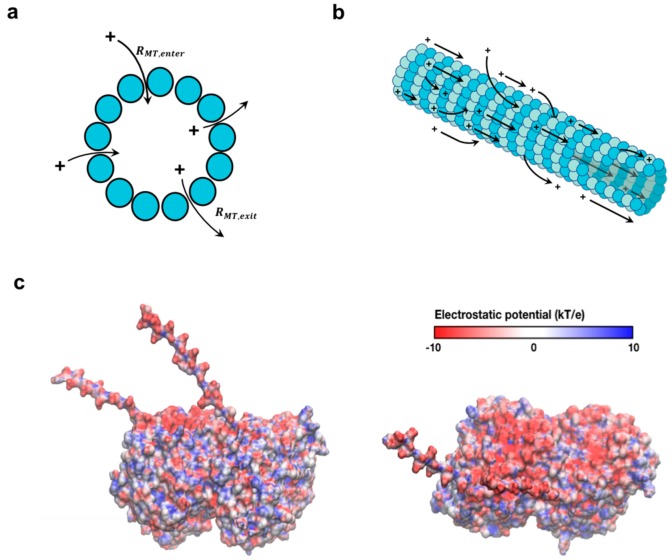
Schematic of charge transport along and across an MT. (**a**) A representation of charge flow across the MT cross section through nanopores present between adjacent protofilaments. (**b**) A representation of charge flow through both inner and outer modes along an MT. Arrows depict charge flow via both mechanisms, enabling MT charge storage across a broad spectrum of frequencies, and charge transport at low AC frequencies in the cell. (**c**) Side view (left) and top view (right) of the tubulin dimer, displaying distribution of electrostatic potential at different locations. The negatively charged C-termini face towards the solution and contains ~50% of the total negative charge on a tubulin dimer.

An increase in capacitance arises from dense counterion condensation on the MT surface. This has been extensively predicted and simulated to arise from a variety of sources [7,8,44,47,48]. First, the negative charge of the tubulin dimer attracts counterions in solution, leading to the presence of a double layer and depletion region outside the microtubule surface [7,8,46,48]. The charge distribution in the MT protein wall is also highly non-uniform, with the outer surface containing approximately four times the charge compared to the inner surface [47] (Figure 9c). This asymmetry between the inner and outer electrostatic potentials serves to enhance capacitance and is responsible for the abnormally large dipole moment of the tubulin dimer [1]. The asymmetry also manifests through C-terminal ‘tails’ composed of 10–12 amino-acids, that can extend 4–5 nm outwards from each tubulin monomer. These slender C-termini tails are highly negative, containing about 50% of the charge of the tubulin dimer [49]. As they stretch outwards into the solution in a pH and ionic strength-dependant manner, they increase the effective area of the tubulin dimer and significantly contribute to the overall MT capacitance [7,8].

Coherent oscillations of these C-terminal tails are modelled to generate solitonic pulses of mobile charge along the outer surface of an MT, creating ionic currents along its length [7,44,50]. Ions from the bulk solution are also modelled to be pumped into the hollow MT lumen through nanopores in its wall, resulting in charge accumulation inside the cylindrical MT over time [45]. A recent study using molecular dynamics simulations showed that the permeability of the MT lumen was significantly higher for Na^+^ and K^+^ as opposed to Ca^2+^, allowing for free movement of selective ions into the MT lumen across its porous surface [47]. To the best of our knowledge, our findings are the first to experimentally quantify this resistance encountered by charge flow across the MT cross section. These results implicate not only ionic movement along the microtubule axis, but also across and inside it, enhancing the modelled roles of MTs as complex subcellular nanowires. 

Manning’s theory of polyelectrolyte solutions predicts the conditions for ionic condensation on charged polymer surfaces provided a sufficiently high linear charge density is present on these surfaces creating an ionic concentration depletion area surrounding them [51]. The sum total of the charges on polymer surfaces and the associated counterions decreases to values dependent on the valence of the counterions and the Bjerrum length, which is the distance from the polymer surface at which the Coulomb energy of the screened surface charges equals the thermal energy. The double layer of surface charges and counterions separated by the Bjerrum length can be viewed as having capacitor-like properties. Although the Manning theory was originally developed for such polyelectrolytes as DNA, it was also applied to actin filaments [52] and MTs [53]. For actin filaments, its application explained the observed lossless transmission of electric pulses along the filament lengths. In the case of MTs, it provided a plausible explanation of unusual amplification of injected electrical signals that propagated along these nanowires. The calculated Bjerrum length for MTs was found to be approximately 6.7 × 10^−10^ m [8,50]. Both actin filaments and MTs have been represented in these models by cable equations with effective real and imaginary impedance due to the viscosity of the solution-resisting ionic flows and the capacitive properties of the ionic double layers around the filaments, respectively [52,53]. The capacitance for a single ring of an MT including C-termini was calculated to be approximately 1.3 × 10^−15^ F [8]. When extended to 20 µm, (representative of the length of a single MT for our measurements), the predicted value would be C = 3 × 10^−12^ F, although an experimental confirmation of this prediction is not directly available through our measurements or in any previous work. We note the relatively weak dependence of network capacitance on MT concentration, and assign it to the random spatial locations and directional orientations of MTs in our solution. Indeed, the conductivity of randomly distributed RC networks has been shown to scale weakly with the number of elements in the network [54]. Additionally, qualitative similarities can be found in the models of random resistor and capacitor networks with a frequency-dependent crossover for both conductance and impedance in these networks due to percolation-type conduction [55]. We intend to develop a quantitative model for our experimental observations in a subsequent publication. 

### 4.2. Implications for the Cell 

Our work, which utilizes cell-like tubulin and ionic concentrations for the first time, indicates a cellular role for microtubules as wires that store charge. Neuronal environments where MTs are spontaneously nucleated from free tubulin, such as growth cones, experience large capacitance changes over short bursts of time. This ability significantly impacts the action potentials that are known to depend strongly on the local charge distributions [56]. Additionally, ionic movement across the MT wall enhances their roles as attenuators of local cation distributions. In nonneuronal environments, transient ionic currents around a MT during mitosis could impact MT dynamics and potentially influence the chromosome segregation. Specifically, Ca^2+^ ion storage/flow about an MT triggers its depolymerisation, whereas waves of Mg^2+^ or lowering in the local pH (increasing H^+^) leads to MT stabilisation [57,58]. The attraction of Zn^2+^ or Mn^2+^ ions in the vicinity leads to the formation of two-dimensional tubulin polymers [59,60]. Properties of the cytoplasm such as polarisability and relative permittivity get severely attenuated because of the presence of MTs in the vicinity. Because of the polymerisation state of tubulin-altering solution capacitance, our findings implicate a temporal evolution of capacitance and ionic flows as the ratio of MTs to free unpolymerised tubulin changes [61,62,63]. MT lattice defects, which occur when a tubulin dimer is missing in an MT wall [64,65], cause a large ionic flux to develop at the defect site. Spatiotemporal charge distribution shifts are also critical at the MT end, where fluxes form because of sudden changes due to the polymerisation/depolymerisation of the MT. Free/polymerised tubulin hence regulates local and global electrical properties, creating spatially dynamic gradients of charge storage and flux. We envision a cytoskeleton that, in addition to transporting macromolecules, stores and transports ionic signals and electrical information across the cytoplasm (Figure 9a,b). 

Our findings can be coupled with a vast array of bio-nanodevices that utilises MTs and MAPs (microtubule-associated proteins) for construction of bio-nanotransporters and bio-actuators [66,67,68,69,70]. Under specific conditions, MAP-MT systems are capable of repositioning macromolecules [71,72], directionally transporting microtubules [15,73] and even drive their movement within zero-mode waveguides [74] and inorganic nanotubes [75]. Storage of electrical charge and its transport along MTs can be coupled to such cutting-edge mechanical MAP-based devices to develop a wide range of nano-actuators and nano-sensors. 

When compared to cells, the rates of MT nucleation and polymerisation are significantly lower in BRB80. This difference can be attributed to the absence of MAPs and macromolecular crowding [76,77]. Mammalian cells contain high concentrations of K^+^ ions (140–300 mM) [27,28], which, in addition to MAPs and molecular crowding agents, will be included in a future study to attain physiological equivalence. We also note that the effect of PTMs (post-translational modifications) on the electrical properties of microtubules has not yet been explored. PTMs involve the addition of residues such as phosphate and glutamate that locally influence counterionic condensation around the outer microtubule surface.

We are in the process of performing DC (direct-current) measurements, determine the contribution of MTs to impedance relaxation time and evaluate the voltage dependence of capacitance on MT-containing solutions. Interestingly, this aspect has been discussed previously: the inductance of a single protofilament is calculated to be <1 fH [8]. Further investigation is required to experimentally confirm these predictions. 

## 5. Conclusions

We used EIS to compare the complex impedance of MT- and tubulin-containing solutions. A physiologically relevant, high ionic strength buffer (BRB80) created a high noise, low impedance background, which was countered through the use of physiological concentrations of tubulin. While the presence of MTs increased solution capacitance, unpolymerised tubulin did not have any appreciable effect. In a study that is the first of its kind to the best of our knowledge, we determined the capacitance and resistance of the MT network at physiological tubulin concentrations to be 1.27 × 10^−5^ F and 9.74 × 10^4^ Ω. These values correspond to an effective resistance per unit volume of 3.71 × 10^10^ Ω/L and effective capacitance per unit volume of 7.65 F/L. We envision a dual electrical role for MTs in the cell, that of charge storage devices across a broad frequency spectrum (acting as storage locations for ions), and of charge transporters (bionanowires) in the frequency region between 20 and 60 Hz. Our findings also indicate that the electrical properties of tubulin dimers change as they polymerise, revealing the potential impact of MT nucleation and polymerisation on the cellular charge distribution. Our work shows that by storing charge and attenuating local ion distributions, microtubules play a crucial role in governing the bioelectric properties of the cell. 

## Figures and Tables

**Table 1:** Volumes of tubulin and buffer solution (BRB80T or BRB80C) used to stabilise microtubules (BRB80T) or free tubulin (BRB80C) in solution.

Tubulin Concentration (μM)	Volume of BRB80T or BRB80C (μL)	Tubulin Volume (μL)
0.222	99.5	0.5
2.222	95	5
22.225	5	5

**Table 2:** Fit parameters attained by fitting the real and imaginary components of impedance to Equation (7). Fit parameters represent effective capacitance *C_MT_*, and resistance *R_MT_* introduced into the solution through the addition of the MT network at different concentrations. *R_H_* is the small constant resistance that is ascribed to small fraction of unpolymerised tubulin that is present in MT-containing solutions. *γ_R_* and *γ_C_* describe the effective resistance and capacitance per unit volume introduced by the microtubule network. *δ R_MT_*, *δ C_MT_* and *δ R_H_* correspond to 95% confidence intervals for the fit parameters. The values *δ γ_R_* and *δ γ_C_* correspond to the uncertainties in the resistance and capacitance per unit volume. Corresponding graphs are displayed in Figure 8.

[Tub] (µM).	C_MT_ (F).	*δC_MT_* (F)	*R_MT_* (Ω)	*δR_MT_* (Ω)	*R_H_* (Ω)	*δR_H_* (Ω)	*γ_R_* (Ω/L)	*γ_C_* (F/L)	*δγ_R_* (Ω/L)	*δγ_C_* (F/L)
22.222	1.27 × 10^−5^	1.48 × 10^−7^	9.74 × 10^4^	1.18 × 10^4^	2.12	40.61	3.71 × 10^10^	7.65	4.49 × 10^9^	0.056
2.222	1.25 × 10^−5^	1.67 × 10^−7^	1.00 × 10^5^	1.40 × 10^4^	0.61	34.79	3.81 × 10^10^	4.76	5.33 × 10^9^	0.063
0.222	2.01 × 10^−5^	3.38 × 10^−7^	9.97 × 10^4^	2.82 × 10^4^	0.41	31.95	3.80 × 10^10^	4.83	1.07 × 10^10^	0.12

## Supplementary Materials

The following are available online at https://www.mdpi.com/2079-4991/10/2/265/s1. Figure S1. 3D printed holders used to fabricate and align the parallel-plate contact device. (**a**) Top view (left) and (**b**) side view (right) of holder for the parallel plate device used to perform impedance measurements. (**c**) Top view (left) and side view (right) of slider used to position the double-sided tape exactly to fabricate the device. (**d**) Top view (left) and side view (right) of the holder used to position the upper contact precisely on the lower contact. Figure S2. Validation of parallel-plate contact device using 0.5 mM electrolytic solutions. (**a**) Imaginary component of impedance for electrolytic solutions at 0.5 mM and de-ionised water. (**b**) Real component of impedance for electrolytic solutions at 100 mM and de-ionised water. Data display average values collected between 15 and 21 times. Error bars represent standard deviation. Figure S3. Example of microtubule and tubulin subtraction with backgrounds to display typical impedance. Figure S4. No ‘reversal’ in the resistive behaviour of microtubules is observed between 10 and 100 Hz. Graphs showing differences in the real component of impedance as a function of decreasing input AC frequency at total tubulin concentrations of (**a**) 22.225 µM, (**b**) 2.222 µM, (**c**) 0.222 µM, (**d**) comparison of the effect of paclitaxel and colchicine on impedance. Figure S5. One-sample t-tests were performed to determine if the impedance difference values were significantly above zero. This was carried out using the t-test function (‘ttest’) within MATLAB. Graphs showing the variation of obtained p-values for the imaginary components of impedance in (**a**) tubulin and (**b**) MT-containing solutions. Graphs showing the variation of obtained p-values for the real components of impedance in (a) tubulin and (b) MT-containing solutions.
