# Supplementary material for: Investigation of the Electrical Properties of Microtubule Ensembles under Cell-Like Conditions
Source: Nanomaterials (Basel). 2020 Feb 5;10(2):265. doi: 10.3390/nano10020265 (PMC7075204; doi:10.3390/nano10020265)
Supplement: Supplementary file 1 [file nanomaterials-10-00265-s001.pdf]

ELECTRONIC SUPPLEMENTARY INFORMATION

Article

Investigation of the Electrical Properties of Microtubule Ensembles under Cell-like Conditions

Aarat P. Kalra[1], Sahil D. Patel[2], Asadullah F. Bhuiyan[2], Jordane Preto[1], Kyle G. Scheuer[2], Usman Mohammed[3], John D. Lewis[4], Vahid Rezania[3], Karthik Shankar[2,*] and Jack A. Tuszynski[1,4]

[1] Department of Physics, University of Alberta, 11335 Saskatchewan Dr NW, Edmonton, Alberta T6G 2M9, Canada; aarat@ualberta.ca (A.P.K.); preto@ualberta.ca (J.P.)

[2] Department of Electrical and Computer Engineering, University of Alberta, 9107–116 St, Edmonton, Alberta T6G 2V4, Canada sdpatel@ualberta.ca (S.D.P.); abhuiyan@ualberta.ca (A.F.B.); scheuer@ualberta.ca (K.G.S.); kshankar@ualberta.ca (K.S.)

[3] Department of Physical Sciences, MacEwan University, Edmonton, Alberta, T5J 4S2, Canada. mohammedu@mymacewan.ca (U.M.); rezaniav@macewan.ca (V.R.)

[4] Department of Oncology, University of Alberta, Edmonton, Alberta, T6G 1Z2, Canada. jdlewis@ualberta.ca (J.D.L.); jackt@ualberta.ca (J.A.T.)

* Correspondence: aarat@ualberta.ca (A.P.K.)

Received: 4 December 2019; Accepted: 29 January 2020; Published: date

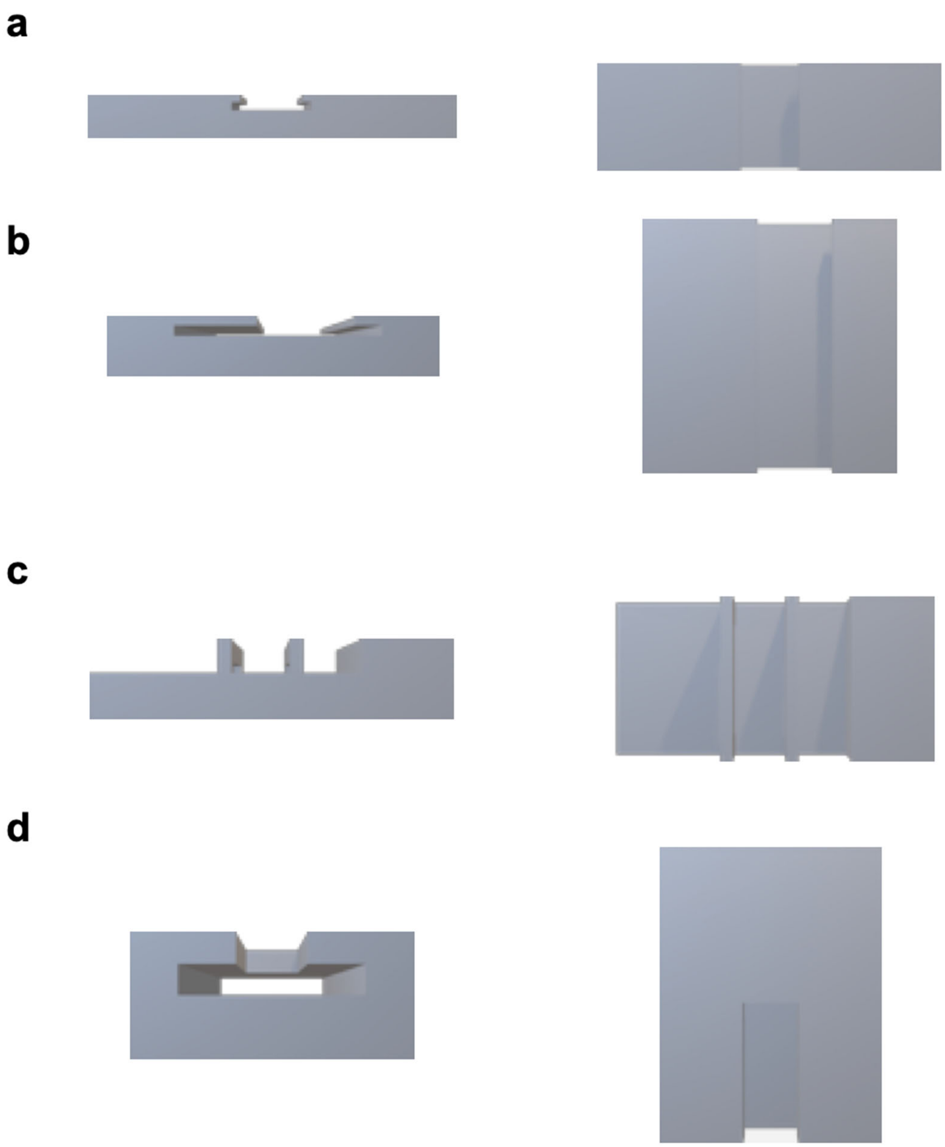

Figure S1. 3D printed holders used to fabricate and align the parallel-plate contact device. **(a)** Top view (left) and **(b)** side view (right) of holder for the parallel plate device used to perform impedance measurements. **(c)** Top view (left) and side view (right) of slider used to position the double-sided tape exactly to fabricate the device. **(d)** Top view (left) and side view (right) of the holder used to position the upper contact precisely on the lower contact.

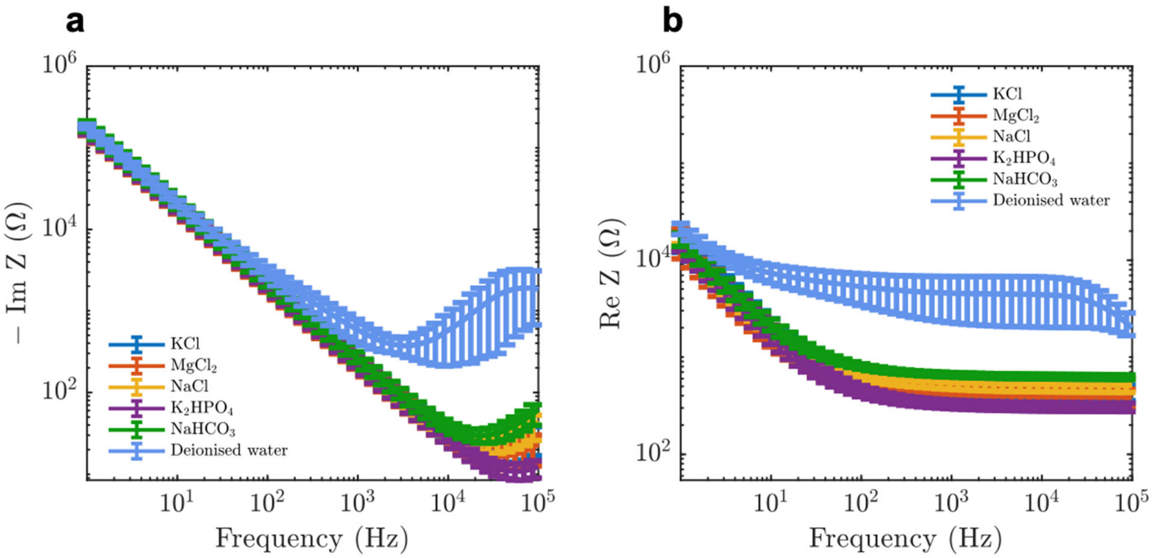

Figure S2. Validation of parallel-plate contact device using 0.5 mM electrolytic solutions **(a)** Imaginary component of impedance for electrolytic solutions at 0.5 mM and de-ionized water. **(b)** Real component of impedance for electrolytic solutions at 100 mM and de-ionized water. Data displays average values collected between 15 and 21 times. Error bars represent standard deviation.

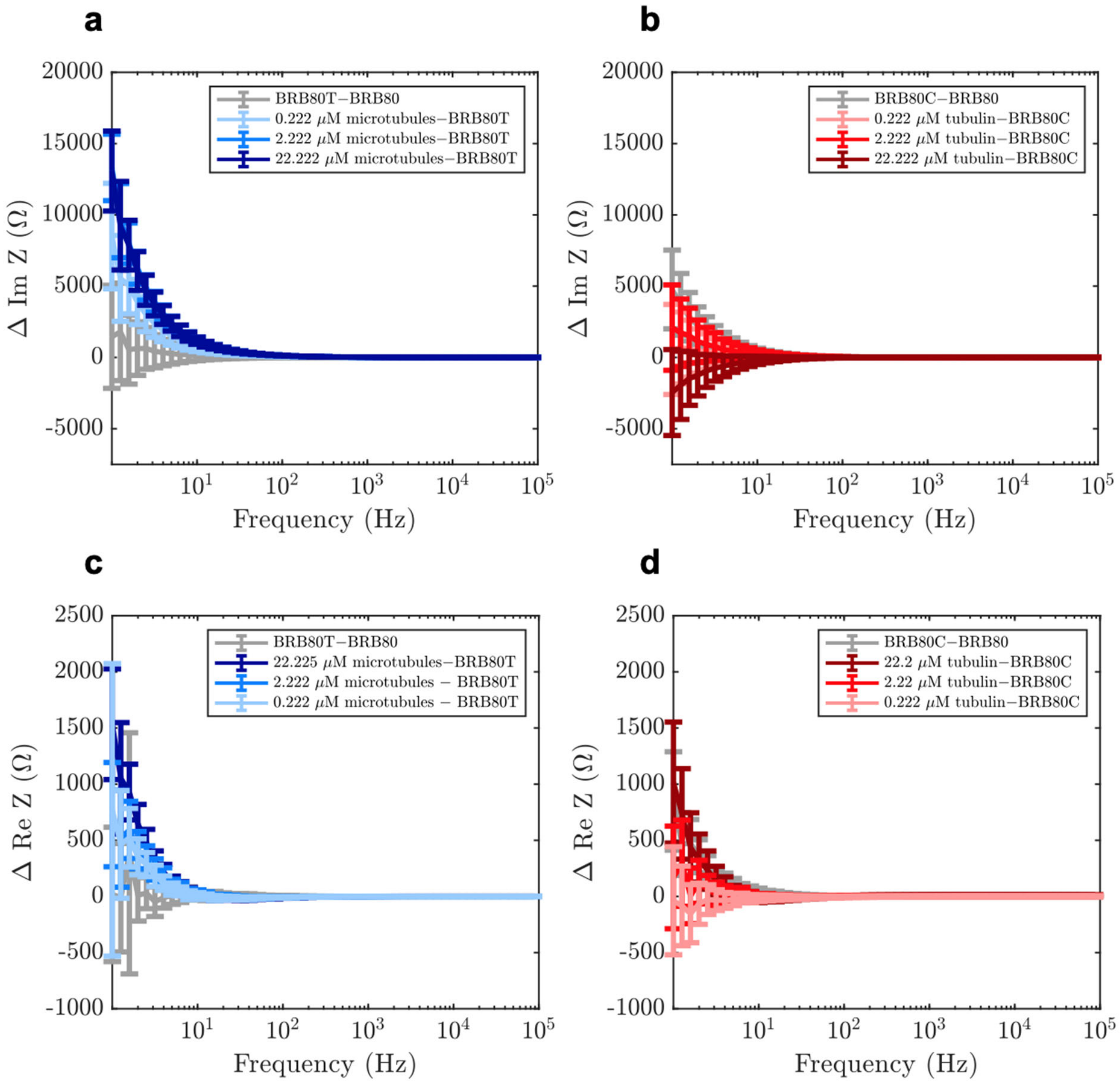

Figure S3. Example of microtubule and tubulin subtraction with backgrounds, to display typical impedance.

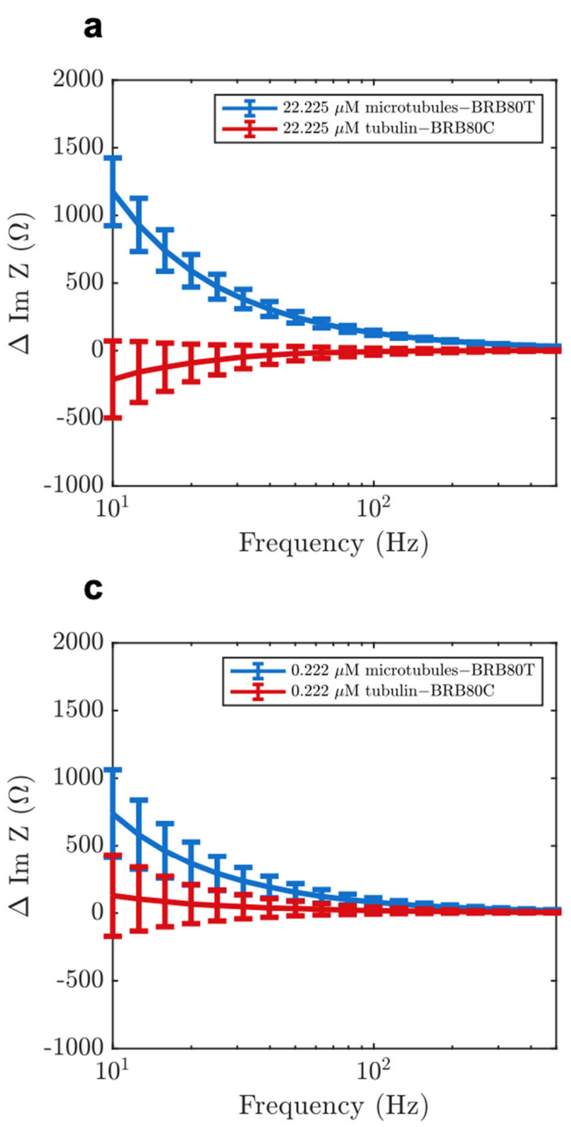

Figure S4. No 'reversal' in the resistive behavior of microtubules is observed between 10 and 100 Hz. Graphs showing differences in the real component of impedance as a function of decreasing input AC frequency at total tubulin concentrations of **(a)** 22.225 μM, **(b)** 2.222 μM, (c) 0.222 μM, **(d)** comparison of the effect of paclitaxel and colchicine on impedance.

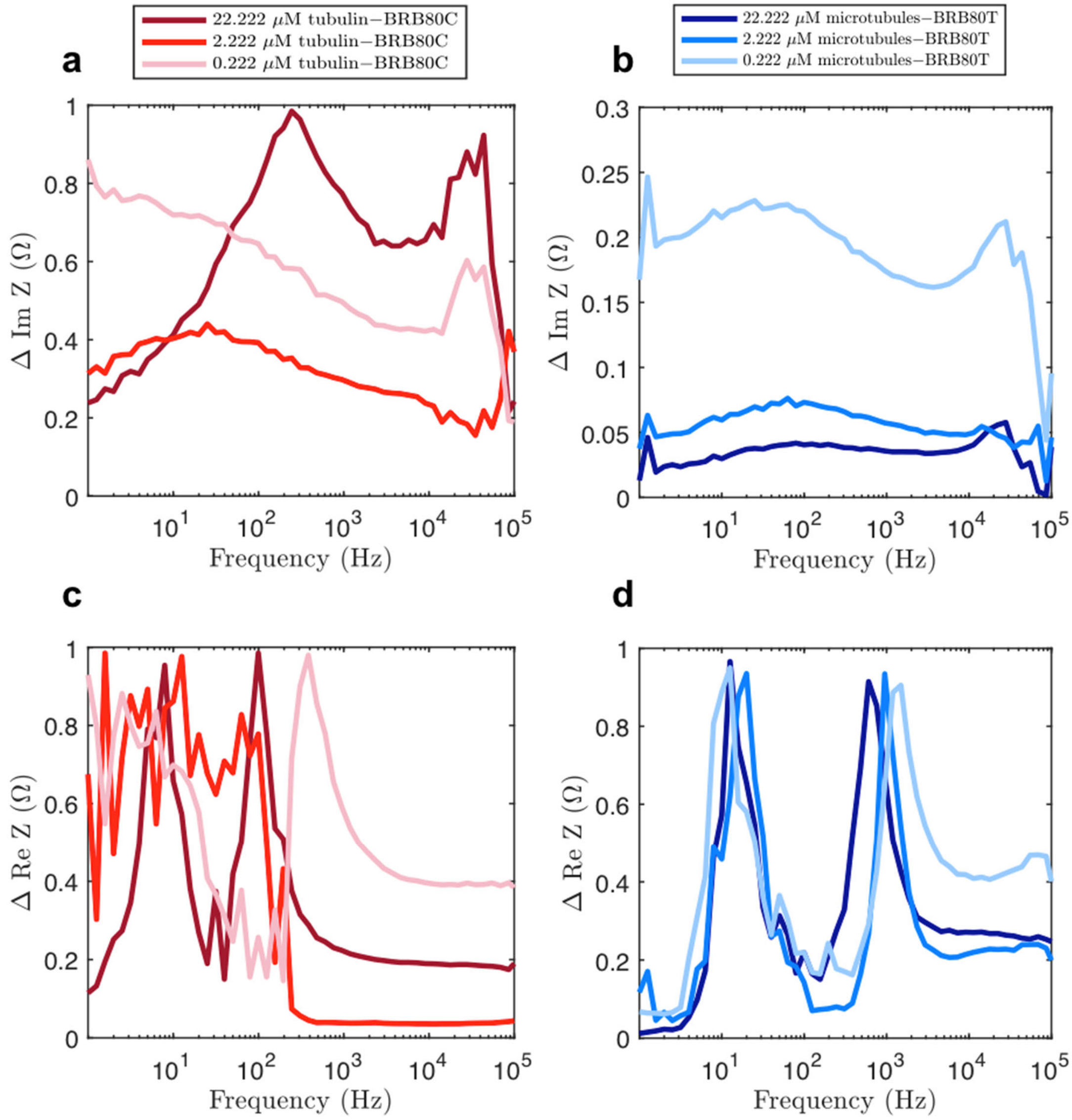

Figure S5. One-sample t-tests were performed using to determine if the impedance difference values were significantly above zero. This was carried out using the ttest function within MATLAB. Graphs showing the variation of obtained p-values for the imaginary components of impedance in (a) tubulin and (b) MT-containing solutions. Graphs showing the variation of obtained p-values for the real components of impedance in (a) tubulin and (b) MT-containing solutions.
